# Supplementary material for: Deletion of wfs1 Impairs Oligodendrocyte Precursor Cells Dorsal Distribution and Myelination Through the wfs1-hmgcs1 Axis in Zebrafish
Source: Biology (Basel). 2026 Aug 16;15(16):1402. doi: 10.3390/biology15161402 (PMC13510151; doi:10.3390/biology15161402)
Supplement: Supplementary file 1 [file biology-15-01402-s001.zip › File S1-Transcriptome sequencing data and differentially expressed genes (DEGs) in wfs1-- zebrafish at 5 dpf/2.KEGG/wfs1KOvsWT/DOWN/src/dre04916.html]

dre04916


- dre:393760

- Down regulated genes

393760\_1(-1.85238730761533)

- dre:58072

- Down regulated genes

58072(-6.00118378322126)
- dre:30369

- Down regulated genes

30369\_1(-1.70732701220414)

- dre:100333318

- Down regulated genes

100333318(-1.02298964704357)

- dre:497384

- Down regulated genes

497384\_1(-2.05267237809407)

- dre:393760

- Down regulated genes

393760\_1(-1.85238730761533)

- dre:556087

- Down regulated genes

556087\_1(-6.16688081226611)
- dre:791993

- Down regulated genes

791993(-3.69340367298196)
- dre:30127

- Down regulated genes

30127\_1(-1.48241226857528)

Close
